# Scrambled Eggs: Apoptotic Cell Clearance by Non-Professional Phagocytes in the *Drosophila* Ovary

**DOI:** 10.3389/fimmu.2017.01642

**Published:** 2017-11-29

**Authors:** Sandy B. Serizier, Kimberly McCall

**Affiliations:** ^1^Department of Biology, Boston University, Boston, MA, United States; ^2^Graduate Program in Molecular Biology, Cell Biology and Biochemistry, Boston University, Boston, MA, United States

**Keywords:** cell death, apoptosis, engulfment, phagocytosis, efferocytosis, epithelial cells, *Drosophila*, oogenesis

## Abstract

For half of a century, it has been known that non-professional phagocytes, such as fibroblasts, endothelial, and epithelial cells, are capable of efferocytosis (engulfment of apoptotic cells). Non-professional phagocytes differ from professional phagocytes in the range and efficiency of engulfment. Much of the recognition and underlying signaling machinery between non-professional and professional phagocytes is the same, but it is not known how the engulfment capacity of non-professional phagocytes is controlled. Moreover, the signaling networks involved in cell corpse recognition, engulfment, and phagosome maturation are only partially understood. The *Drosophila* ovary provides an excellent system to investigate the regulation of phagocytic activity by epithelial cells, a major class of non-professional phagocytes. During *Drosophila* oogenesis, mid-stage egg chambers undergo apoptosis of the germline in response to nutrient deprivation. Epithelial follicle cells then undergo major cell shape changes and concomitantly engulf the germline material. Our previous work has established that Draper and the integrin α-PS3/β-PS heterodimer are required in follicle cells for germline cell clearance. In addition, we have characterized phagosome maturation pathways, and found that the JNK pathway amplifies the engulfment response. In this review, we discuss recent advances on the interplay between engulfment pathways in the follicular epithelium for cell clearance in the *Drosophila* ovary. We also provide a comparison to apoptotic cell clearance mechanisms in *C. elegans* and mammals, illustrating strong conservation of efferocytosis mechanisms by non-professional phagocytes.

## Introduction

Apoptotic cell clearance by phagocytic cells is critical for organismal homeostasis. Professional phagocytes are cells whose main task in the milieu is to efficiently clear dead cells. Non-professional phagocytes, on the other hand, have other tissue-resident functions, but can engulf when needed. Differences between professional and non-professional phagocytes are not well understood. In this review, we present the *Drosophila* ovary as an outstanding model to investigate engulfment by non-professional phagocytes. We first discuss the diversity of apoptotic cell clearance pathways across *Drosophila, C. elegans*, and mammals. We next discuss professional and non-professional phagocytes in different organisms with an emphasis on the molecular biology of apoptotic cell clearance in the *Drosophila* ovary by epithelial follicle cells. We compare the follicle cell model to examples of phagocytosis by epithelial cells in mammals and their clinical relevance to health and disease.

## Apoptotic Cell Clearance Mechanisms in *C. elegans, D. melanogaster*, and Mammals

The CED-2, -5, and -12 and CED-1, -6, and -7 pathways were first identified in *C. elegans* as the major pathways that control engulfment. Both pathways act in parallel and converge on CED-10 (Rac1) to promote the cytoskeletal rearrangements required for engulfment (1). Rho family GTPases, such as Rac1 and Cdc42, function downstream of apoptotic cell recognition to induce cytoskeletal shape changes to form a phagocytic cup. Rac1 functions across all model systems and is the best characterized cytoskeletal modulator of engulfment (2–4). In *C. elegans*, CDC42 acts in parallel to CED-2/5/12 and CED-10 and downstream of CED-1/6/7 (5). The first *cell death abnormal* (*ced*) genes in *C. elegans, ced-1* and *ced-2*, were identified in a screen for mutants with an abnormal persistence of embryonic cell corpses. While all corpses are cleared in late stages of embryogenesis in wild-type strains, *ced-1* and *-2* mutants have persisting corpses (6). Ellis et al. later conducted a mutagenesis screen to isolate maternal effect mutations that prevent corpse clearance. In this screen, analysis of CED mutant progeny of egg laying defective mothers found additional alleles of *ced-1* and *-2*, and identified *ced-5, -6, -7, -8*, and *-10* genes as regulators of corpse clearance (7). Electron microscopy revealed that these mutants specifically exhibit defects in engulfment at the uptake step. Double mutant analysis determined that CED-2, -5, and -12 and the CED-1, -6, and -7 signaling axes act in parallel.

One of the earliest experiments that supported the conservation of apoptotic cell clearance mechanisms across organisms was a study whereby the expression of human orthologs was shown to rescue the CED mutant clearance defects. Specifically, Dock180, the mammalian ortholog of CED-5, was shown to be capable of rescuing the *ced-5* mutant phenotype (8). These early studies in *C. elegans* complemented the discovery of signaling machinery that control apoptotic cell clearance in mammals (1, 8–14).

The engulfment machinery in *C. elegans* is conserved in mammals (Table 1). Mammalian Multiple EGF-Like Domains 10 (MEGF-10) is homologous to CED-1, a transmembrane receptor that binds to phosphatidylserine, an aminophospholipid that is exposed on the surface of apoptotic cells and functions as an “eat me” signal (15). The immunoreceptor tyrosine-based activation motifs (ITAMs) of MEGF-10 are phosphorylated by the Src family kinases, and this mediates interaction with Syk tyrosine kinase for the activation of downstream effectors. Engulfment Adaptor PTB Domain Containing 1 (GULP), the CED-6 ortholog, is an adaptor protein that binds to the NPXY motif of the intracellular domain of MEGF-10 *via* its PTB binding domain (12, 16). ABCA1/7, the CED-7 ortholog, is an ABC transporter that has been shown to function in both the apoptotic and engulfing cell. ABCA1 has recently been shown to function in homeostasis to increase cholesterol efflux during apoptotic cell clearance by macrophages (17). CrkII (CED-2 ortholog), Dock180 (CED-5 ortholog), and ELMO (CED-12 ortholog), all encode cytoplasmic signaling proteins that help propagate the engulfment process by activating Rac1 (CED-10 ortholog). The SH3 domain of Dock180 interacts with the PxxP motif and PH domain of ELMO. This ELMO contact with Rac1 and Dock180 stabilizes the Rac1/Dock180 interaction, allowing for Rac1 activation (17). The functional contribution of Cdc42 in mammals is more elusive and context dependent. Specifically, dominant negative Cdc42 blocks F-actin recruitment to phagocytic cups in BMM and NIH3T3 cells (18, 19), but has no effect on photoreceptor outer segment uptake in the retinal pigment epithelium (20). Surprisingly, overexpression of Cdc42 does not induce more phagocytosis in NIH3T3 cells (19). Several additional engulfment receptors have been identified in mammals including BAI1, Tim4, Stablin-2, and MERTK (21–25).

**Table 1:** Engulfment machinery in professional and non-professional phagocytes in *C. elegans, D. melanogaster*, and mammals.

	*C. elegans*	*D. melanogaster*	Mammals
Engulfment receptors	CED-7 αPAT-3/INA-1 CED-1 C03F11.3 ND ND ND ND ND ND ND ND ND ND	ND αPS3βPS/βν Draper Croquemort NimC4/SIMU ND ND ND ND ND ND ND ND ND	ABCA1/7 αvβ5/β3 Integrin Megf10/SCARF1 CD36 ND BAI-1 BAI-3 TIM-1 TIM-4 MerTK Fc Receptor Stablin 2 LRP/1 KIM-1
Adaptor proteins	CED-2 CED-5 CED-6 CED-12	ND Mbc/Sponge Ced-6 Ced-12	CrkII Dock180 GULP ELMO 1(2)
GTPases	Ced-10 Cdc-42	Rac1 Rac2 Cdc42	Rac1 Cdc42

*Orthologs based on literature review. ND indicates ortholog not determined*.

Like mammals, conservation of the underlying engulfment machinery has also been demonstrated in *Drosophila* (Table 1). For example, Draper, the *Drosophila* ortholog of MEGF-10/CED-1, requires Src42A (Src ortholog) and Shark (Syk ortholog) kinase activity for the clearance of severed axons in the brain, similar to mammals (26, 27). *Drosophila* glia also require Crk/Mbc (CED-2/CED-5) for the clearance of axonal debris (28). Crk and Mbc, but not ELMO/Ced-12, are required downstream of integrins αPS3/ßν, for apoptotic cell priming for efficient engulfment by hemocytes (29). Studies in *Drosophila* hemocytes have uncovered new players in engulfment such as Pallbearer, an E3-ubiquitin ligase, ribosomal protein S6, and Rac2 (30). In addition, Undertaker, a junctophilin, responds to calcium flux to mediate clearance by hemocytes (31). Many engulfment genes have been shown to have other functions in *Drosophila*. For example, Mbc and Ced-12/ELMO are required for ommatidial development, myoblast fusion, and cell migration (32–38).

## Professional and Non-Professional Phagocytes in *C. elegans, D. melanogaster*, and Mammals

Professional phagocytes, such as macrophages and monocytes, function to maintain tissue homeostasis by removing dying and infected cells from the milieu (39). Professional phagocytes engulf with high efficiency and have been shown to use several engulfment receptors to complete the task (17, 40–43). Integrins, CD36, and MEGF-10 have been shown to function in professional phagocytes (Table 1) (44–47). In *Drosophila*, hemocytes are macrophage-like cells that circulate to clear apoptotic cells and pathogens (48–50). They have also been shown to be critical for proper patterning of the central nervous system, innate immunity, and wound healing (51). Similar to mammals, multiple receptors and bridging proteins, including Draper, Six-microns-under, Croquemort, and Integrins function in apoptotic cell clearance by hemocytes.

Non-professional phagocytes have other resident functions, but can engulf when needed. Examples of non-professional phagocytes include epithelial and endothelial cells, and astrocyte glia (Table 2). Interestingly, *C. elegans* do not have professional phagocytes and, therefore, rely solely on neighboring non-professional phagocytes to clear apoptotic debris. The diverse engulfment receptors used by professional phagocytes for clearance of apoptotic cells are conserved in non-professional phagocytes (16). In *Drosophila*, several cell types, including epithelial follicle cells and imaginal disk cells have been shown to function as non-professional phagocytes (52, 53). Thus, like mammals, *Drosophila* utilizes both professional and non-professional phagocytes.

**Table 2:** Examples of non-professional phagocytes in *C. elegans, D. melanogaster*, and mammals.

*C. elegans*	*D. melanogaster*	Mammals
Hypodermal cells Gonadal sheath cells Pharyngeal muscle cells Endothelial cells	Follicle cells Ensheathing glia Cortex glia Astrocyte glia Imaginal disks Epidermal cells	Mammary gland Retinal pigment epithelium Gut epithelium Liver endothelium Airway epithelium Kidney epithelium

The mechanisms by which professional and non-professional phagocytes communicate and coordinate their activities are currently under investigation. In mammals, alveolar macrophages release IGF-1 when mice are exposed to house dust mites and IGF-1 activates IGF-1R on the surface of airway epithelial cells. This interaction functions to redirect airway epithelial cells from engulfing to initiating an inflammatory response (54). It also stimulates airway epithelial cells to takeup macrophage-derived multivesicular bodies (MVBs) that contain anti-inflammatory cytokines. The anti-inflammatory cytokine containing MVBs suppress the expression of pro-inflammatory gene PTX3, suggesting a mechanism whereby macrophages signal to non-professional phagocytes to resolve inflammation (55).

## The *Drosophila* Ovary as a Model for Studying Non-Professional Phagocytes

**Figure 1:**
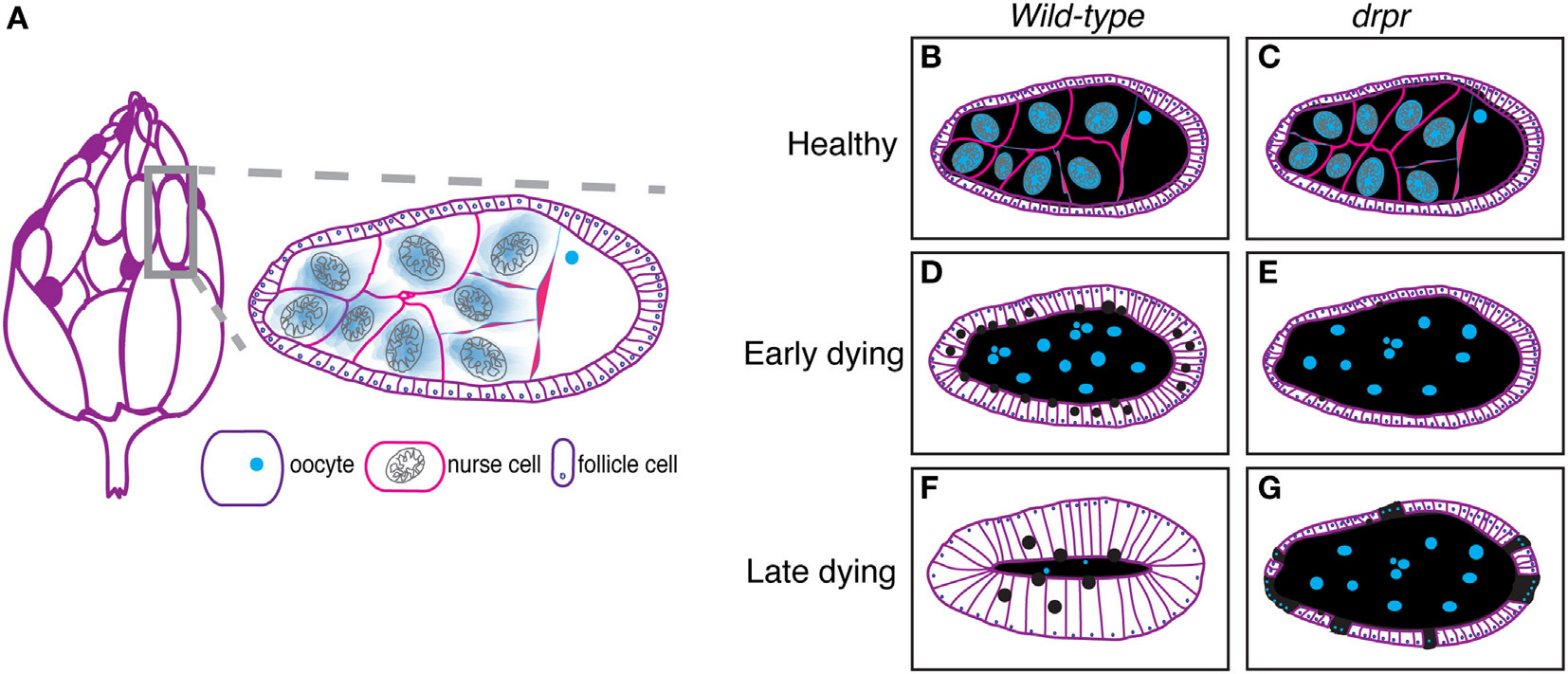
The *Drosophila* ovary as a model for engulfment. **(A)** Each ovary (left) comprises strands of developing eggs called egg chambers. Stage 8 egg chamber (gray box) zoom (right) has three main cell types: germline-derived nurse cells and oocyte and somatically derived follicle cells. Dark purple egg chambers (left) correspond to the egg chambers that spontaneously die in the ovary. Ovary drawing adapted from Ref. (52). **(B–G)** Schematics of healthy and degenerating egg chambers in wild-type and *draper*^Δ^ *^5^* mutant flies. Cell membranes are shown in purple, nuclei in blue, nurse cell cytoplasm in black, and follicular epithelium in white. Wild-type starvation-induced apoptotic nurse cells are cleared by enlarged follicle cells **(D)** until there is little to no germline material left **(F)**. *draper* mutant follicle cells do not enlarge and fail to clear germline material **(E)**. Follicle cells prematurely die (cyan dots) **(G)**.

The *Drosophila* ovary (Figure 1A, left) comprises 20 strands of progressively developing egg chambers called ovarioles. Egg chambers arise from the germarium, the anteriormost region of each ovariole that houses germline and follicle stem cells. The germline stem cells produce cystoblasts that undergo four rounds of mitosis to generate cysts containing 16 interconnected cells. Due to incomplete cytokinesis of the dividing cysts, each egg chamber is arranged in a syncytium, where each germline cell is connected to the next through ring canals. One of the 16 cyst cells is specified as the oocyte and the remaining 15 cells differentiate into polyploid nurse cells, whose main task is to provide organelles, RNA, proteins, and nutrients necessary for oocyte growth and embryogenesis. During the division, specification, and differentiation steps, the germarium produces somatically derived epithelial follicle cells that surround the 15 nurse cells and oocyte, which together constitute an egg chamber (Figure 1A, right, Figures 1B,C). The epithelial follicle cells serve as a protective barrier for the growing germline. Follicle cells also synthesize and secrete yolk and proteins that mediate the formation of vitelline membrane and chorion. As described below, the follicle cells also act as non-professional phagocytes.

Each egg chamber progresses through 14 well-characterized stages of oogenesis (56). Vitellogenesis begins at stage 8 of oogenesis, which has a characteristic loss of proportion between nurse cells and oocyte, where the oocyte becomes noticeably larger than the nurse cells. During vitellogenesis, the follicle cells begin to synthesize and transport yolk proteins into the oocyte. Oocyte growth culminates in a process called dumping, whereby the nurse cells rapidly dump all of their cytoplasmic contents into the oocyte, leaving little nurse cell cytoplasm behind (57). Nurse cells then undergo programmed cell death with nuclear condensation, fragmentation, acidification, and clearance by the follicle cells.

## Checkpoints of Cell Death in the Ovary

Aside from the developmental cell death that naturally occurs in all egg chambers at the end of oogenesis, insults such as starvation have been shown to induce cell death earlier in oogenesis. These cell deaths occur in response to checkpoints during specific stages of oogenesis when the tissue senses and responds to environmental changes. The earliest checkpoint occurs in the germarium and the second occurs in mid-oogenesis at the onset of vitellogenesis between stages 7 and 9. It is thought that mid-stage egg chambers are primed to respond to environmental stimuli before investing in the energetically expensive vitellogenic process (58, 59).

Several factors have been shown to induce cell death at mid-oogenesis, including temperature, mating, daylength, developmental abnormalities, chemical treatment, cocaine exposure, cellular phone irradiation, and starvation, but only starvation-induced cell death mechanisms are well characterized (59–64). Apoptosis, the best characterized type of programmed cell death, is defined by caspase activation, chromatin condensation, DNA fragmentation, membrane blebbing, cell shrinkage, and the formation of apoptotic bodies (65). Nurse cell death in mid-oogenesis has been shown to be apoptotic using TUNEL assays to detect fragmented DNA, active effector caspase Dcp-1/Casp3 staining, and DNA morphology analysis that detects highly condensed chromatin (60, 61, 66, 67). The canonical apoptotic pathway in *Drosophila* consists of the activation of pro-apoptotic proteins Reaper, Hid, and Grim (RHG). RHG proteins facilitate activation of an apoptosome-like complex (involving Dark and Dronc) and inactivation of the anti-apoptotic protein Drosophila inhibitor of apoptosis 1 (Diap-1), which leads to activation of effector caspases (Drice and Dcp-1) to dismantle the cell (68). Unusually, nurse cells in mid-oogenesis use mechanisms independent of RHG proteins and the apoptosome to initiate death (69). What is known during the mid-oogenesis checkpoint is that the effector caspase Dcp-1 is essential. Rather than undergoing apoptosis, nurse cell nuclei of Dcp-1 null or Diap-1 overexpressing flies fail to condense, and surrounding follicle cells prematurely die (67, 70, 71).

Buszczak et al. proposed that ecdysone-responsive genes function as part of a surveillance mechanism to detect environmental conditions before progressing to later stages of oogenesis (72). In support of this hypothesis, there are several lines of evidence that ecdysteroid signaling determines whether an egg chamber will progress past mid-oogenesis. Ecdysteroid concentrations increase in ovary extracts upon starvation (73). Early ecdysone genes are expressed in follicle cells at the developmentally sensitive mid-oogenesis timepoint (72, 74, 75). *E75^e213^* germline mutant clones arrest at stages 8 and 9 of oogenesis, and adrenodoxin reductase, the enzyme required for steroid hormone synthesis, is required in the germline for egg chambers to progress beyond vitellogenic stages (72).

## Characterization of Engulfment by Epithelial Follicle Cells

Germline cell death in mid-oogenesis is coupled with engulfment by the surrounding follicle cells, providing a powerful model for engulfment by epithelial cells. Engulfment by follicle cells was first visualized by electron microscopy (52), and later by observations of uptake of fluorescent germline markers (71, 76). In an effort to closely investigate the morphological changes that take place during engulfment by the follicle cells, Etchegaray et al. characterized engulfment in response to starvation-induced apoptosis (77). They found that the underlying follicular epithelial cells synchronously enlarged to engulf the apoptotic germline and the growth in the follicle cells correlated with nurse cell nuclear condensation and fragmentation (Figures 1B,D,F). The epithelial follicle cells proceeded to engulf the apoptotic germline until there was no material left (Figures 1D,F).

**Figure 2:**
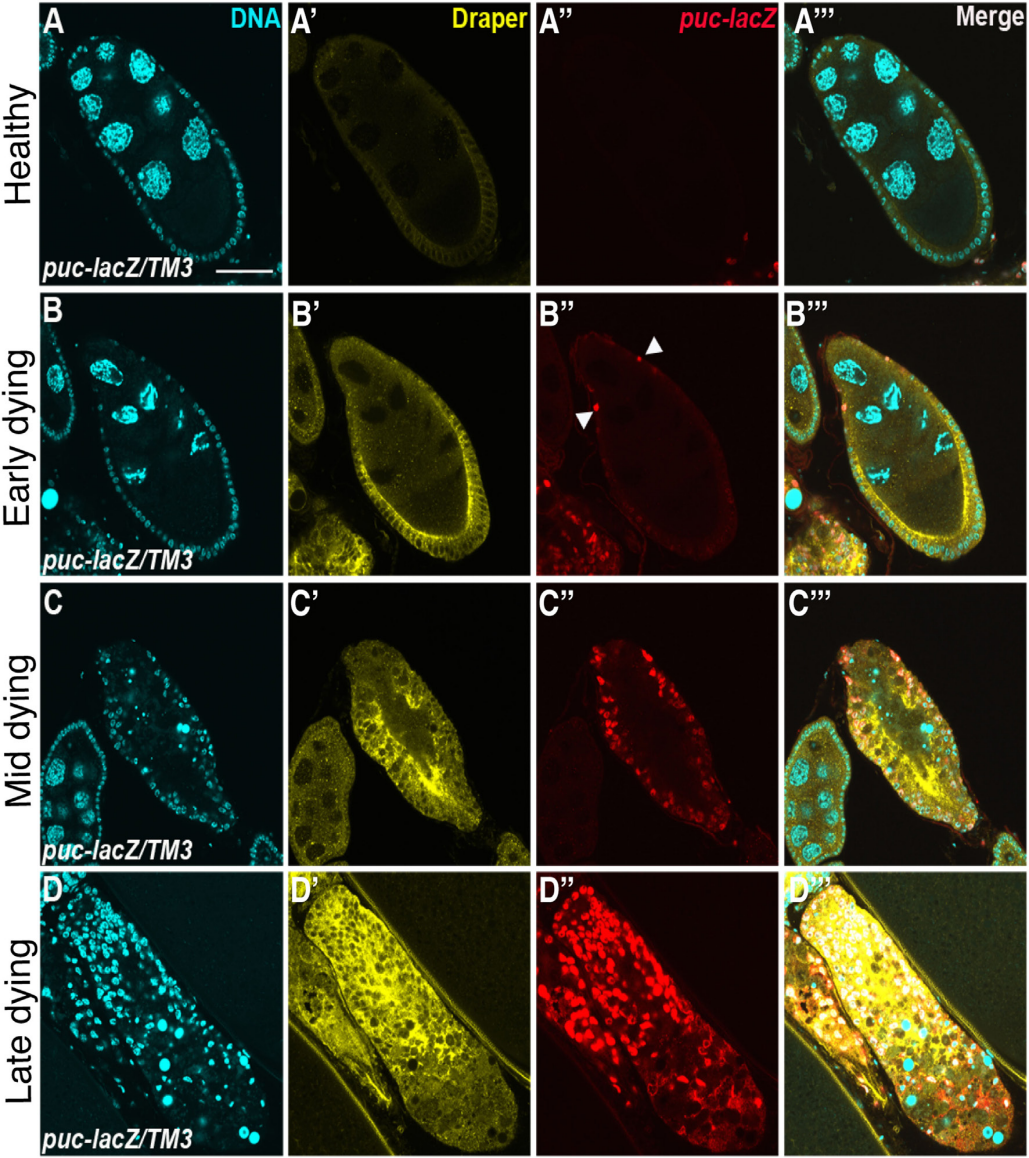
Draper and the JNK signaling pathway are activated in follicle cells during engulfment. Healthy **(A)** and degenerating **(B–D)** egg chambers expressing the JNK reporter *puc-lacZ*, stained with anti-Draper (yellow), anti-βgal (red) and DAPI (cyan). Draper is expressed at low levels in healthy egg chambers **(A′)** and increases in expression throughout the progression of engulfment **(B′–D′)**. *puckered* is not expressed in healthy egg chambers **(A″)**, but is activated during engulfment **(B″–D″)**, merged images are shown in panels **(A′″–D′″)**. Adapted with permission from Ref. (77).

Draper, the *Drosophila* CED-1 ortholog, had been shown to be required for engulfment in multiple tissues, and it was also found to be required in the follicle cells. Draper protein expression levels increases throughout the progression of engulfment in mid-oogenesis (Figure 2), indicating that follicle cells may modulate their phagocytic capacity by Draper upregulation (77). *draper* null mutants have severe defects in the uptake of germline material, showing a lack of follicle cell enlargement, premature follicle cell death, and apoptotic germline material that fails to be cleared [Figures 1E,G; (77)]. Draper RNAi knockdown in the epithelial follicle cells, but not the germline showed the same phenotype, and overexpression of *draper* in the follicle cells in the *draper* null mutant background rescues these defects, demonstrating that Draper is required in the underlying follicular epithelium for clearance of the dying germline.

To determine the mechanism by which Draper engulfs the apoptotic germline, candidate screens were conducted to identify other genes that are required for Draper-mediated engulfment (77–79). Draper-associated Shark kinase propagates intracellular signaling by recognizing and binding to the phosphorylated YXXL motif of the intracellular domain of Draper (27). Engulfment by follicle cells fails to proceed in the absence of Shark or Src42A, another Draper receptor kinase (78). CED-6 function has been implicated in *C. elegans* and mammals as an adaptor molecule, but RNA interference that targets *Ced-6* in follicle cells has no phenotype, suggesting that another adaptor may function in this context or RNAi knockdown was incomplete (80). Sponge, one of the *Drosophila* CED-5 orthologs, and Ced-12 have both been confirmed to be required for engulfment by follicle cells (78, 80). Consistent with engulfment pathways in other tissues and organisms, expression of dominant negative Rac1, the mediator of cytoskeletal shape changes required for engulfment, blocks engulfment by epithelial follicle cells.

The integrin α-PS3/β-PS heterodimer is induced and required for engulfment by the follicular epithelium during starvation-induced germline death (78). One might speculate that integrins function in tandem with Draper in follicle cells for efficient clearance of apoptotic germline corpses. The following hypotheses could explain the need for multiple receptors: (1) one receptor may function to signal downstream to activate Rac1, while the other receptor functions as a tethering molecule by binding “eat me” signals on the apoptotic cell surface; (2) both receptors may function to activate downstream signals, which converge at Rac1 for efficient engulfment; and (3) both receptors may function to activate downstream signals, one of which will converge onto Rac1 and another that has other cellular functions. α*-PS3 draper* double mutants manifested more severe engulfment defects than single mutants, but were not completely defective in engulfment, which suggests that other receptors may contribute to this engulfment process. Mutant analyses of Croquemort, a scavenger receptor previously implicated in cell clearance and known to be expressed in the ovary, were also investigated in combination with Draper and integrins, but did not worsen the defects in engulfment (78), which suggests another engulfment receptor may function in the ovary. The engulfment machinery required for clearance by the folllicular epithelium in the Drosophila ovary and *C. elegans* and mammalian orthologs are listed in Table 3.

**Table 3:** Required engulfment machinery in *Drosophila* follicular epithelium and orthologs.^a^

*D. melanogaster*	Mammals	*C. elegans*
Eiger Draper Integrin α-PS3 Integrin β-PS Crumbs Mekk1 Basket Kayak Cka Shark Shibire Deep orange Sponge Ced-12 Rac1 Dhc64C Bazooka aPKC Par-6 Cdc42	TNF-α Megf-10 Integrin subunit α4^a^ Integrin subunit β1^a^ Crumbs 1 MAP3k4 MAPK8 Fos ND Syk Dynamin 1 Vps18 Dock-3^a^ ELMO Rac1 Dync1h1 PARD3 PRKCI Par-6 Cdc42	ND CED-1 ND ND CRUMBS-1 MTK-1 JNK-1 FOS-1 ND ND DYNAMIN-1 VPS18 CED-5 CED-12 CED-10 DHC-1 PAR-3 PKC-3 PAR-6 CDC-42

*^a^Orthologs were identified using the DRSC Integrative Ortholog Prediction Tool*.

*ND indicates ortholog not determined*.

## Apoptotic Cell Clearance is Affected by Cell Polarity

Based on observations of enriched expression of Draper and integrins on the apical surface of the follicular epithelium, Meehan et al. (78) sought to determine whether proper cell polarity was required for engulfment. *aPKC, baz, par-6, crb*, and *Dhc64C* were all found to be required for the progression of engulfment, suggesting that an underlying directionality is required for proper localization of engulfment receptors. Indeed, *Dhc64C* (Dynein heavy chain) knockdown in follicle cells by RNAi blocks α-PS3/β-PS apical enrichment. Moreover, *aPKC* knockdown in follicle cells prevents Draper enrichment to the apical surface, suggesting a novel mechanism whereby polarity genes regulate localization of multiple engulfment receptors in epithelial cells. Cdc42 is also required for the progression of engulfment in the *Drosophila* ovary. Follicle cells of engulfing egg chambers form a double layer at the posterior end, similar to cell polarity mutant follicle cells (78, 79), suggesting that Cdc42 functions in cell polarity.

The follicle cell model system shows interesting similarities to the mammalian retinal pigment epithelium. Mouse integrin αv, β5, MFGE8, and MERTK mutants exhibit defects in the clearance of shed outer segments of the retina (81–85). Mouse myosin VIIa mutants have abnormal apical localization of engulfed phagosomes in retinal pigment epithelial cells, suggesting that cell polarity or phagosome trafficking contributes to outer segment disk clearance (86).

## Phagosome Maturation in the *Drosophila* Ovary

Phagosome maturation is the last step of corpse clearance where the apoptotic corpse is internalized into a phagosome, or vesicle that matures to its final degradation. Membrane modifications change throughout the maturation process and can be used as markers to visualize how far along the corpse is in the steps of degradation. The earliest modification known to occur is an increase in PtdIns(4,5)P_2_ in early phagocytic cups (87). As the phagocytic cup progresses, PtdIns(4,5)P is depleted and PtdIns(3,4)P_2_ and PIP3 concentrations increase (87). Much of what is known about phosphoinositol changes during phagosome maturation has been characterized in Fc-receptor-mediated clearance by macrophages (88–90). In *C. elegans*, the sequence of protein recruitment is conserved whereby PtdIns3P and DYNAMIN-1 function at the phagocytic cup followed by RAB2, RAB5, and RAB7 recruitment and fusion with lysosomes (91–95). Upon internalization, the phagosome transitions into an increasingly acidic organelle and fuses with lysosomes to complete corpse degradation. In addition to lipid modifications, the phagosome first associates with RAB5 GTPase and as the phagosome matures, RAB5 is replaced by RAB7. RAB7 is finally replaced by LAMP-1, targeting the corpse for degradation *via* lysosomal fusion (96). PtdIns3P, Rab5, and Rab7 phagosome maturation markers have all been found to occur in the same sequence in the *Drosophila* ovary. *draper*^Δ^*^5^* mutants were found to not only have defects in uptake, as shown by a reduced number of engulfed Dcp-1 positive particles, but also could not process the little material engulfed, as shown by the lack of the Rab7 phagosome maturation marker and LysoTracker-positive vesicles compared to controls. *Ced-12* and *Src42A* mutants exhibited fewer vesicles taken up and matured, but there were only mild defects in acidification. α-PS3 mutants had defects in uptake, but Dcp-1 positive vesicles that were able to form, did get coated with Rab7, and were acidified. These findings suggest that the α-PS3/β-PS integrin heterodimer is required for the uptake of dying germline corpses, but Draper has a dual requirement for uptake and phagosome maturation (78).

## Draper Signals to the JNK Signaling Pathway in Many *Drosophila* Tissues

**Figure 3:**
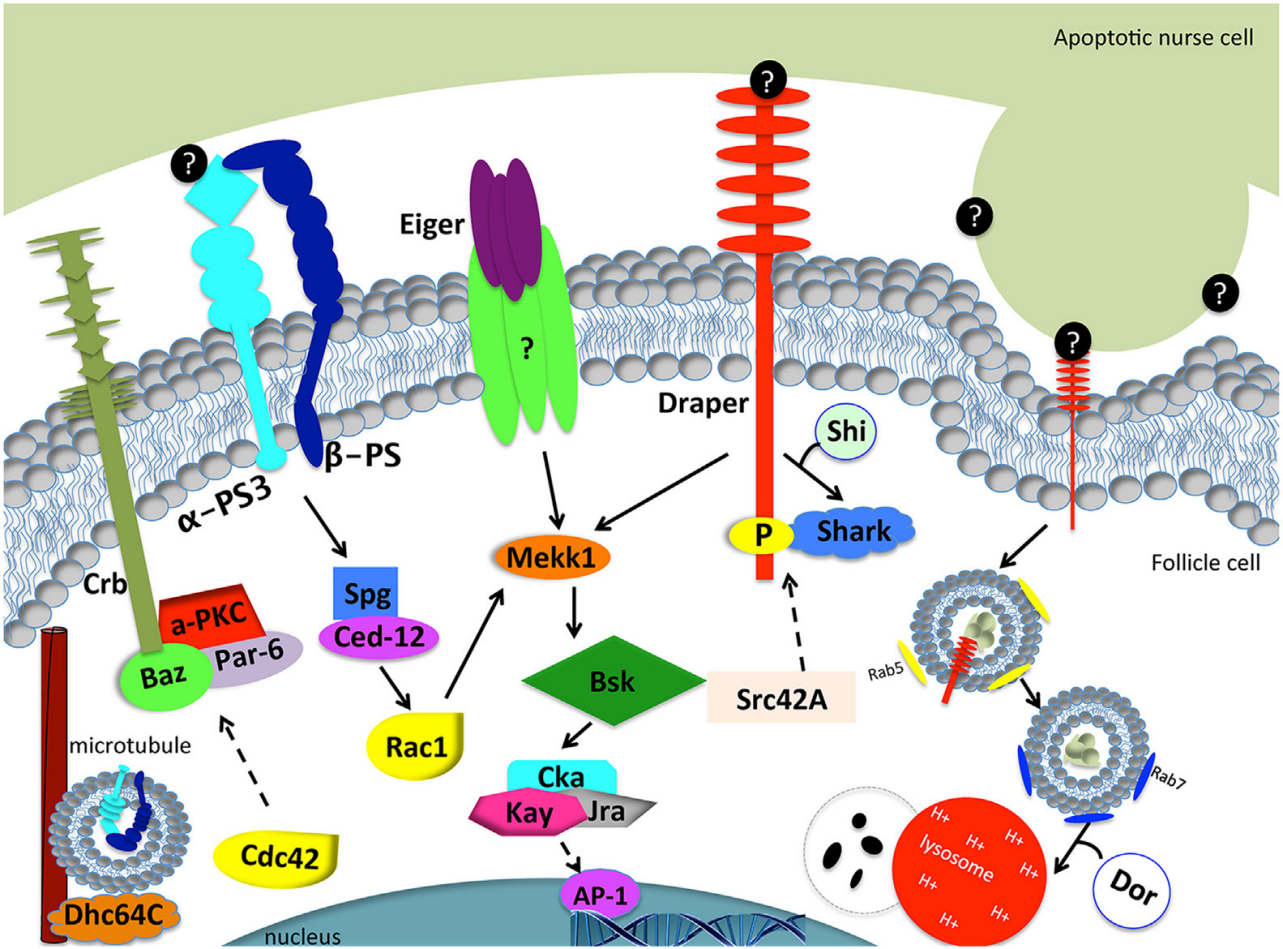
Model of engulfment by follicle cells in the *Drosophila* ovary. Apoptotic cell recognition (left side): Draper binds to an unknown “eat me” signal (black circles) and activates the JNK signaling cascade (Mekk1, Bsk, Cka, Kay, Jra) through Shark to mediate engulfment. Eiger may or may not interact with a TNF receptor to activate the JNK signaling pathway. α-PS3/β-PS integrin heterodimer signals through Spg and Ced-12 to activate Rac1. Rac1 also activates the JNK signaling pathway. The Crb/Baz, Par-6, and aPKC polarity proteins are also required for engulfment. Dhc64C assists in the trafficking of α-PS3/β-PS. Phagosome maturation (right side): Draper is required for nurse cell phagosome maturation. Draper and corpse material first become enclosed in Rab-5 positive phagosomes and mature into Rab-7 positive phagosomes, until the phagosome fuses with lysosomes for degradation. Shi is required for early phagosome maturation. Dor is required for phagosome and lysosome fusion. Dashed arrows correspond to proteins that were tested without epistasis analysis and have engulfment defects. Question marks correspond to unknown proteins.

The JNK signaling pathway is pleiotropic and can induce a variety of downstream effectors that include apoptotic machinery. To determine whether JNK signaling played a role in mid-oogenesis cell death, the expression of *puckered (puc)*, a JNK signaling pathway responsive gene, was investigated (77). Rather than being activated in the dying germline, Puc was specifically induced in follicle cells of apoptotic egg chambers and its expression increased with the progression of engulfment (Figure 2). Knockdown of JNK signaling pathway components *Mekk1* (JNKKK), *Bsk* (JNK), *Jra* (jun), or *kayak* (fos) by RNA interference in the follicle cells results in engulfment defects, demonstrating a requirement for JNK signaling in engulfment in the ovary. However, RNA interference of *hemipterous* (JNKK), *slipper* (JNKKK), or *misshapen* in the follicle cells results in normal engulfment, suggesting that only some of the canonical pathway members are required in this context. Similar to the ovary, *Mekk1* and *kayak* are also required in the adult brain for axonal debris clearance, but in contrast to the ovary, Tak1 and Slipper activate Mekk1 in the brain (97). Furthermore, Misshapen loss of function in the adult brain blocks engulfment progression, suggesting context dependent mechanisms for JNK axis activation (98). The JNKKKK and JNKKK acting in the ovary remain to be determined. One activator of JNK signaling is Eiger, the TNF-α ortholog. Eiger interacts with receptors Wengen and Grindelwald, a recently identified TNFR ortholog (99). Eiger/Wengen interaction results in the activation of Misshapen (Msn, JNKKKK), Tak1 (JNKKK), Hemipterous (Hep, JNKK), and Basket (Bsk, JNK). Eiger loss of function results in defects in engulfment in the ovary, suggesting that Eiger may activate JNK in the follicle cells (77). A summary of the signaling pathways in the follicle cells is shown in Figure 3.

In the ovary, JNK activity is markedly delayed in *draper* null mutants, suggesting that Draper and the JNK cascade act in the same signaling pathway (77). When the JNKK Hep is constitutively expressed in the *draper* null background, engulfment defects are suppressed, suggesting that JNK acts downstream of Draper, and can activate other targets that facilitate engulfment. Activation of *hep* promotes Draper expression, suggesting JNK is required for *draper* induction during engulfment. Consistent with findings in the ovary, studies in the *Drosophila* embryonic central nervous system, adult brain, and wing epithelium have shown a requirement for the JNK pathway downstream of Draper (27, 100, 101). Studies in the embryonic central nervous system show that while expression levels of Draper and the bridging molecule Six-microns-under remain the same in glia that have active JNK signaling, *hep^CA^* gain of function in *draper* loss-of-function mutants restores defects in apoptotic clearance (101). In the adult brain, Traf4, a Misshapen binding partner in the JNK signaling pathway, is required for Draper-mediated JNK activation in glial cells for axonal clearance (102). More recent clonal analyses in the brain revealed that TRE, a JNK signaling reporter, fails to turn on in *draper*^Δ^*^5^* clones compared to wild type, suggesting a cell autonomous requirement for *draper* activation of JNK in glia for clearance of axons in response to axonal injury, like the ovary (102). In macrophages, JNK is also required for the induction of *draper* in response to corpses (103). Surprisingly, overexpression of Draper II, the inhibitory isoform of Draper, results in increased JNK pathway activity in the wing (100). Taken together, these studies in *Drosophila* indicate that Draper and JNK regulate each other in multiple cellular contexts. JNK is activated in professional and non-professional phagocytes in mammals (104, 105), but whether JNK is required remains to be determined. Perhaps JNK signaling machinery is a prerequisite for phagocytes to increase phagocytic capacity.

Apoptotic cells are thought to signal to phagocytes for their removal by exposing caspase-dependent “eat me” signals. Dcp-1, an effector caspase, is required for germline cell death in response to starvation in the ovary, and mutants block engulfment progression but surprisingly do not affect Draper localization to the follicle cell membrane or activation of the JNK pathway during engulfment. Moreover, overexpression of Dcp-1 in the germline induces cell death but there is delayed JNK activity and Draper expression in the engulfing epithelial follicle cells. These findings suggest that the caspase Dcp-1 is required for an “eat me” signal that acts independently of Draper and JNK. Eat me signals from the germline have not been identified.

## Murderers by Nature: Death by Phagoptosis

Interestingly, Draper overexpression in epithelial follicle cells was found to induce nurse cell death in the absence of starvation (77). Egg chambers induced to die by *draper* expression have an underlying epithelium with active JNK signaling, the key regulator of engulfment in starvation-induced cell death. This suggests that engulfment machinery has the ability to induce the death of an otherwise healthy cell, a form of cell death coined “phagoptosis” (106). The defining characteristic of phagoptosis is that the loss of function of engulfment machinery blocks cell death. Other characteristics of phagoptosis include the induction of “eat me” signals or the loss of “don’t eat me” signals on the surface of healthy cells. Intriguingly, *draper* loss-of-function mutants show a delay in subtle aspects of germline death, including chromatin fragmentation and loss of the oocyte nuclear lamina (80, 107), suggesting that Draper has a normal function in promoting death of the germline. Indeed, Draper directly contributes to cell death during nurse cell developmental cell death in late oogenesis (108).

In *C. elegans*, it has long been thought that engulfment machinery contributes to cell death (109). Engulfment mutants show enhanced survival of cells destined to die in a *ced* mutant background (109, 110). Interestingly, CED-1 has been shown to promote asymmetric localization of the caspase CED-3 in mothers of apoptotic cells (111). Another example where phagocytic machinery is required for death in *C. elegans* occurs in the developing male tail during larval stages (112). In mammals, phagoptosis has been reported in several processes, such as blood cell clearance (113–115), which allows for rapid turnover of blood cells during tissue homeostasis. During mammalian eye development, macrophages induce phagoptosis of vascular endothelial cells by locally releasing WNT7b ligands (116, 117). This newly studied cell death mechanism has implications in therapy, as a recent study found that malignant B cell cancer cell lines die by phagoptosis (118). Furthermore, amyloid β induces superoxide release from microglia to murder neurons (119). Characterization of the mechanisms of phagoptosis may help understand how cancer cells evade death and how amyloid β leads to neurodegeneration.

Fly examples of phagoptosis have also come to light. Kuraishi et al. modified the ER retention motif of Pretaporter, a Draper “eat me” signal, to contain the sequence of a glycosylphosphatidylinositol anchor, which artificially induced Pretaporter exposure on the surface of healthy cells (120). They found that this exposure increases the phagocytic capacity of engulfing S2 cells. This result suggests that the exposure of an “eat me” signal on an otherwise healthy cell can result in its degradation, supporting the conservation of phagoptosis mechanisms across organisms. Interestingly, cell competition studies in wing imaginal disks determined that Draper, Wasp, Mbc/Dock180, and Rac1 engulfment proteins are required for the elimination of adjacent Minute mutant cells (121). *scrib* and *dlg*-induced imaginal disk tumors are eliminated by the activation of a JNK/PVR/Mbc engulfment signaling axis in wild-type adjacent cells (53). These examples indicate that phagoptosis might be more widespread than previously thought.

## Epithelial Cell Mechanisms of Engulfment Across Organisms

For many years, it has been thought that non-professional phagocytes could not clear apoptotic cells from tissue with the same efficiency as professional phagocytes (122). For example, macrophage-free mice have mesenchymal cells that engulf to a lesser extent (123). Microglia need to contact an apoptotic cell once to initiate clearance, but non-professional phagocytes can take hours to respond post-recognition (124). This suggests that non-professional phagocytes require more time to assemble engulfment machinery and may not be primed to the same extent as professional phagocytes. Studies in kidney 293 cells, however, have shown that the efficiency in the ability to clear apoptotic cells may not always be the result of the type of phagocyte that clears, but the mechanism by which apoptosis is induced (125).

Many non-professional phagocytes are epithelial cells that are found in a wide range of tissues of the human body, including the mammary gland, gastrointestinal tract, and eye (16). Mammary epithelial cells play a key role in phagocytosis during involution post-lactation where mammary alveolar epithelial cells undergo apoptosis and are cleared by adjacent alveolar epithelial cells and macrophages (126). Defects in apoptotic mammary alveolar epithelial cell clearance coincide with inflammation that results in inefficient redevelopment of mammary glands (127). Mfge-8, Dock180, and Rac1 are all required for alveolar epithelial cell death and clearance (127, 128). Although Monks et al. (129) could not detect macrophages or neutrophil presence during involution, other immunohistochemistry and microarray experimental findings demonstrate a change in macrophage gene expression profile changes in mice post weaning (129–132). When macrophages are depleted from mice prior to milk fat globule production for weaning, mammary epithelial cell death, adipocyte repopulation, and other postpartum involution events fail to occur, which suggests that macrophages contribute to the signaling that drives these events post weaning (133).

An example of phagocytic epithelial cells in the eye are the retinal pigment epithelial cells (RPE), a single layer of epithelial cells housed on the retina that line the outer segment of the eye between the photoreceptor cells and choroid. RPE have microvilli that project into the outer segment layer to pinch off and clear the shed distal outer segment ends of photoreceptor cells as part of the daily circadian schedule. Daily clearance of turned over outer segment prevents oxidative toxicity by photoreceptor cells and is critical for vision. Many receptors are required for clearance by RPE including integrins, CD36, and MERTK (81, 85).

A critical role for phagocytic epithelial cells in the colon was recently shown in a mouse dextran sodium sulfate (DSS)-induced colitis mouse model where BAI1 mRNA loss positively correlates with disease progression (134). Members of the TAM engulfment receptor family, interestingly, are either lowly expressed or when expressed are incapable of compensating for the increase in disease progression of BAI1 null mutants. Reintroduction of recombinant BAI1 to DSS-treated BAI1 null mice resulted in a decrease in colitis severity index. Strikingly, BAI1 overexpression in colon epithelium, but not in the myeloid cell lineage, alleviates colitis disease progression. This suggests a critical role for non-professional phagocytes in the colon. This study has shed light on some of the local tissue-specific contributions of epithelium independent of professional phagocytes and may help reveal other disease contexts where professional phagocytes are incapable of clearing tissue secondarily to engulfment defects in the milieu (134).

Asthma patients typically have excessive apoptotic airway epithelial cells in their mucus. Although professional macrophages, such as macrophages, neutrophils, and dendritic cells, circulate in the bronchus, because epithelial cells are in close proximity to apoptotic airway cells, Juncadella et al. asked whether the engulfment machinery of airway epithelial cells contribute to the clearance of apoptotic cells. Indeed, Rac1 loss of function in tracheal and lung epithelium in airway allergen- exposed mice led to increased inflammation and mucus buildup reminiscent of asthma (16, 135). Airway epithelial cells can clear apoptotic epithelial cells and function to dampen the inflammation caused by allergen exposure. These studies illustrate that non-professional phagocytes have a greater function in apoptotic cell clearance and relevance to human disease than previously appreciated.

## Open Questions in the Field about Apoptotic Cell Clearance

While much progress has been made in understanding the molecular biology of apoptotic cell clearance, much is still unknown. Many systems of engulfment across model systems require multiple engulfment receptors for cell clearance to occur properly, but little is known about why more than one is necessary for the execution of clearance. In cases where non-professional phagocytes rapidly remove apoptotic cells, how do they propagate the signal to macrophages once the task is under control? Furthermore, there are many examples where both professional and non-professional phagocytes contribute to apoptotic cell clearance, but how do these two cell populations communicate to ensure that the apoptotic cells are cleared and inflammation is dampened? Draper has been shown to function in multiple steps of engulfment in follicle cells. What signaling mechanisms allow for this molecular switch allowing for Draper to not only initiate cytoskeletal shape changes but also promote apoptotic corpse maturation to lysosomes?

Non-professional phagocytes need to undergo molecular changes that allow them to increase their phagocytic capacity for apoptotic cell clearance. It would be of interest to determine how epithelial cells enhance their phagocytic capacity and whether the JNK signaling pathway promotes such capacity. Moreover, how the Rho family GTPases affect polarity during engulfment is unknown. Whether all epithelial cells require underlying polarization for enhanced phagocytic capacity is also of interest. Interestingly, when follicle cells cannot engulf, they prematurely die. What signaling cues are responsible for premature follicle cell death and what are the environmental cues that control follicle cell death? Expression of Draper in follicle cells promotes germline cell death. What are the mechanisms of phagoptosis in the absence of nutrient deprivation? What factors does Draper rely on to induce apoptotic versus non-apoptotic cell death? The *Drosophila* ovary is an exceptional model for understanding engulfment by non-professional phagocytes and can be used to address pressing questions in the field.

## Author Contributions

SS prepared the figures. SS and KM wrote the manuscript.

## Conflict of Interest Statement

The authors declare that the research was conducted in the absence of any commercial or financial relationships that could be construed as a potential conflict of interest.
